# Porifera of Greece: an updated checklist

**DOI:** 10.3897/BDJ.4.e7984

**Published:** 2016-11-01

**Authors:** Eleni Voultsiadou, Vasilis Gerovasileiou, Nicolas Bailly

**Affiliations:** ‡ Department of Zoology, School of Biology, Aristotle University of Thessaloniki, Thessaloniki, Greece; § Institute of Marine Biology, Biotechnology and Aquaculture, Hellenic Centre for Marine Research, Heraklion, Greece

**Keywords:** Sponges, Demospongiae, Homoscleromorpha, Calcarea, Hexactinellida, Aegean Sea, Sea of Crete, Levantine Sea, Ionian Sea, Eastern Mediterranean

## Abstract

**Background:**

The checklist of Porifera of Greece was created in the framework of the Greek Taxon Information System (GTIS), an initiative of the LifeWatchGreece Research Infrastructure (ESFRI) that has resumed efforts to compile a complete checklist of species recorded from Greece. An updated checklist of Porifera was created on the basis of a list of the Aegean Demospongiae and Homoscleromorpha published one decade ago. All records of species known to occur in Greek waters were taxonomically validated and cross-checked for possible inaccuracies and omissions. Then, all recent publications were reviewed and the species recorded from 2006 to date were added to the list.

**New information:**

The updated checklist of Porifera of Greece comprises 215 species, classified to 111 genera, 65 families, 24 orders, and 4 classes. In total, 34 new additions were made to the previous species list (8 Calcarea, 17 Demospongiae, 1 Hexactinellida, and 6 Homoscleromorpha) with Calcarea being listed for the first time from the area. The demosponge orders Poecilosclerida, Dictyoceratida, Tetractinellida, Haplosclerida, and Suberitida have the highest number of species covering 62% of the known Greek sponge species richness. It is worth mentioning that 8 species have been first described from Greek waters, 7 of which are considered endemic to this area. Our bibliographic overview also revealed knowledge gaps with regard to specific habitats typically rich in sponge diversity, and marine sectors of Greece.

## Introduction

The history of sponge science is directly linked to Greek civilization, since the older written references to sponges are found in Homer’s Epics, and their scientific knowledge has been established by the Greek philosopher, and first marine biologist, Aristotle in his zoological works (Voultsiadou 2007).

In modern times, research on Porifera of the Greek seas started early in the 20^th^ century with the study of bath sponges, i.e., the members of the family Spongiidae (Szymanski 1904, Arndt 1937). In the subsequent decades, up to the 1980s, a series of scattered records of sponge species followed, which can be traced either in faunistic papers (Pérès and Picard 1958, Tortonese 1947) or in more general works on Porifera (Topsent 1920, Vacelet 1969, Griessinger 1971, Pulitzer-Finali 1983).

The systematic research on Greek Porifera started in the 1990s when the Laboratory of Zoology, Aristotle University of Thessaloniki, presented a series of publications on the Aegean sponge taxonomy, ecology and biogeography. New species were described (Voultsiadou-Koukoura and van Soest 1991a, Voultsiadou-Koukoura and van Soest 1991b, Voultsiadou-Koukoura et al. 1991), species lists presented (Voultsiadou-Koukoura and Koukouras 1993, Voultsiadou-Koukoura and van Soest 1993), and the associations of sponges with other invertebrates investigated (Koukouras et al. 1996 and references therein). A checklist of all Aegean sponge species reported up to 2005 and an overview of the relevant literature was provided by Voultsiadou (2005a) and followed by two publications on the distribution of Aegean and Levantine Porifera in the Mediterranean context (Voultsiadou 2005b, Voultsiadou 2009).

The recent study of sponges in the Greek seas comprises taxonomic accounts, including molecular works (Kefalas and Castritsi-Catharios 2007, Kefalas and Castritsi-Catharios 2012, Vacelet et al. 2008, Dailianis et al. 2011), faunistic and ecological papers (Voultsiadou et al. 2010, Voultsiadou et al. 2011, Gerovasileiou and Voultsiadou 2012, Bianchi et al. 2014), and records of species in publications focusing on particular sponge taxa (Ereskovsky et al. 2009).

The aforementioned literature addresses mainly the classes Demospongiae and Homoscleromorpha. No research on Hexactinellida of the Greek seas has been carried out (but see Boury-Esnault et al. 2014), while few species of Calcarea have been recorded mostly in general faunistic publications (e.g. Pansini et al. 2000, Gerovasileiou et al. 2015).

The aim of the present work was to present an updated, annotated checklist of Porifera of the Greek seas. For this purpose, older lists of the classes Demospongiae and Homoscleromorpha were updated according to the recent literature and taxonomic status, and a first attempt to provide a catalogue of Calcarea was made.

## Materials and methods

The Checklist of Porifera of Greece (Suppl. material 1) was created in the framework of the Greek Taxon Information System (GTIS), an initiative of the LifeWatchGreece Research Infrastructure (ESFRI) that has resumed efforts to compile a complete checklist of all species reported from Greece (Bailly et al. 2016). In that publication, a methodology is described to produce Preliminary Checklists only. However, in the present case of Porifera, the status of the list for Greece was quite advanced, and the recent primary literature was exhaustively searched for this work: the present list is thus considered as an updated, annotated, and archived checklist.

The checklist of Demospongiae and Homoscleromorpha was constructed based on a previous inclusive list of the Aegean sponges published by Voultsiadou (2005a). A cross-checking of all species names was carried out and some names were updated according to the World Porifera Database (WPD) (Van Soest et al. 2016) which is part of the World Register of Marine Species (WoRMS) initiative (WoRMS Editorial Board 2016). Four species [i.e. *Cerbaris curvispiculifer* (Carter, 1880), *Oceanapia fistulosa* (Bowerbank, 1873), Petrosia (Strongylophora) vansoesti Boury-Esnault, Pansini & Uriz, 1994, and *Pseudosuberites hyalinus* (Ridley & Dendy, 1886)] were deleted because their Mediterranean records have been considered invalid according to the WPD. Nine of the species mentioned in that list [i.e. *Acarnus tortilis* Topsent, 1892, Clathria (Clathria) coralloides (Scopoli, 1772), *Cliona vermifera* Hancock, 1867, *Cliothosa hancocki* (Topsent, 1888), *Holoxea furtiva* Topsent, 1892, Lissodendoryx (Anomodoryx) cavernosa (Topsent, 1892), *Placospongia decorticans* (Hanitsch, 1895), *Rhabderemia topsenti* van Soest & Hooper, 1993, and *Timea mixta* (Topsent, 1896)] were not included in the present checklist, since they have been to date reported only from the Turkish coast of the Aegean Sea, but not from the Greek waters. Then, all recent publications were reviewed and the species recorded from 2006 to date have been added to the list. The records of freshwater species from Greece were derived from the catalogue published by Pronzato and Manconi (2001).

## Checklists

### Checklist of Porifera known to occur in Greek waters

#### CALCAREA

#### BAERIDA

#### Petrobionidae

#### Petrobiona massiliana

Vacelet & Lévi, 1958

#### CLATHRINIDA

#### Clathrinidae

#### Clathrina clathrus

(Miklucho-Maclay, 1868)

#### Clathrina blanca

(Schmidt, 1864)

#### Leucettidae

#### Leucetta solida

(Schmidt, 1862)

#### LEUCOSOLENIDA

#### Grantiidae

#### Leucandra nausicaae

(Schuffner, 1877)

#### Sycettidae

#### Sycon elegans

(Bowerbank, 1845)

#### Sycon faulkneri

Ilan, Gugel, Galil & Janussen, 2003

#### Sycon raphanus

Schmidt, 1862

#### DEMOSPONGIAE

#### AGELASIDA

#### Agelasidae

#### Agelas oroides

(Schmidt, 1864)

#### Hymerhabdiidae

#### Hymerhabdia intermedia

Sarà & Siribelli, 1960

#### Prosuberites longispinus

Topsent, 1893

#### AXINELLIDA

#### Axinellidae

#### Axinella cannabina

(Esper, 1794)

#### Axinella damicornis

(Esper, 1794)

#### Axinella polypoides

Schmidt, 1862

#### Axinella verrucosa

(Esper, 1794)

#### Heteroxyidae

#### Didiscus stylifer

Tsurnamal, 1969

#### Myrmekioderma spelaeum

(Pulitzer-Finali, 1983)

#### Raspailiidae

#### Eurypon cinctum

Sarà, 1960

#### Eurypon clavatum

(Bowerbank, 1866)

#### Eurypon coronula

(Bowerbank, 1874)

#### Eurypon major

Sarà & Siribelli, 1960

#### Raspaciona aculeata

(Johnston, 1842)

#### Raspailia (Raspailia) viminalis

Schmidt, 1862

#### Stelligeridae

#### Paratimea pierantonii

(Sarà, 1958)

#### Stelligera stuposa

(Ellis & Solander, 1786)

#### BUBARIDA

#### Dictyonellidae

#### Acanthella acuta

Schmidt, 1862

#### Dictyonella incisa

(Schmidt, 1880)

#### Dictyonella marsilii

(Topsent, 1893)

#### Dictyonella obtusa

(Schmidt, 1862)

#### Dictyonella plicata

(Schmidt, 1880)

#### CHONDRILLIDA

#### Chondrillidae

#### Chondrilla nucula

Schmidt, 1862

#### Thymosiopsis cuticulatus

Vacelet & Perez, 1998

#### Halisarcidae

#### Halisarca dujardinii

Johnston, 1842

#### CHONDROSIIDA

#### Chondrosiidae

#### Chondrosia reniformis

Nardo, 1847

#### CLIONAIDA

#### Clionaidae

#### Cliona celata

Grant, 1826

#### Cliona parenzani

Corriero & Scalera-Liaci, 1997

#### Cliona rhodensis

Rützler & Bromley, 1981

#### Cliona schmidtii

(Ridley, 1881)

#### Cliona thoosina

Topsent, 1888

#### Cliona viridis

(Schmidt, 1862)

#### Spiroxya heteroclita

Topsent, 1896

#### Spirastrellidae

#### Diplastrella bistellata

(Schmidt, 1862)

#### Diplastrella ornata

Rützler & Sarà, 1962

#### Spirastrella cunctatrix

Schmidt, 1868

#### DENDROCERATIDA

#### Darwinellidae

#### Aplysilla rosea

(Barrois, 1876)

#### Dendrilla acantha

Vacelet, 1958

#### Dictyodendrillidae

#### Spongionella pulchella

(Sowerby, 1804)

#### DESMACELLIDA

#### Desmacellidae

#### Desmacella annexa

Schmidt, 1870

#### Desmacella inornata

(Bowerbank, 1866)

#### DICTYOCERATIDA

#### Dysideidae

#### Dysidea avara

(Schmidt, 1862)

#### Dysidea fragilis

(Montagu, 1814)

#### Dysidea incrustans

(Schmidt, 1862)

#### Dysidea tupha

(Martens, 1824)

#### Pleraplysilla spinifera

(Schulze, 1879)

#### Irciniidae

#### Ircinia dendroides

(Schmidt, 1862)

#### Ircinia oros

(Schmidt, 1864)

#### Ircinia paucifilamentosa

Vacelet, 1961

#### Ircinia retidermata

Pulitzer-Finali & Pronzato, 1981

#### Ircinia variabilis

(Schmidt, 1862)

#### Ircinia vestibulata

(Szymanski, 1904)

#### Sarcotragus foetidus

Schmidt, 1862

#### Sarcotragus pipetta

(Schmidt, 1868)

#### Sarcotragus spinosulus

Schmidt, 1862

#### Spongiidae

#### Coscinoderma sporadense

Voultsiadou-Koukoura, van Soest & Koukouras, 1991

#### Hippospongia communis

(Lamarck, 1814)

#### Spongia (Spongia) lamella

(Schulze, 1879)

#### Spongia (Spongia) nitens

(Schmidt, 1862)

#### Spongia (Spongia) officinalis

Linnaeus, 1759

#### Spongia (Spongia) virgultosa

(Schmidt, 1868)

#### Spongia (Spongia) zimocca

Schmidt, 1862

#### Thorectidae

#### Cacospongia mollior

Schmidt, 1862

#### Fasciospongia cavernosa

(Schmidt, 1862)

#### Hyrtios collectrix

(Schulze, 1880)

#### Scalarispongia scalaris

(Schmidt, 1862)

#### HAPLOSCLERIDA

#### Callyspongiidae

#### Callyspongia septimaniensis

Griessinger, 1971

#### Siphonochalina expansa

Sarà, 1960

#### Chalinidae

#### Dendroxea lenis

(Topsent, 1892)

#### Haliclona (Gellius) dubia

(Babic, 1922)

#### Haliclona (Gellius) fibulata

(Schmidt, 1862)

#### Haliclona (Gellius) microsigma

(Babic, 1922)

#### Haliclona (Halichoclona) fulva

(Topsent, 1893)

#### Haliclona (Halichoclona) perlucida

(Griessinger, 1971)

#### Haliclona (Haliclona) simulans

(Johnston, 1842)

#### Haliclona (Reniera) aquaeductus

(Schmidt, 1862)

#### Haliclona (Reniera) cinerea

(Grant, 1826)

#### Haliclona (Reniera) cratera

(Schmidt, 1862)

#### Haliclona (Reniera) mediterranea

Griessinger, 1971

#### Haliclona (Reniera) subtilis

Griessinger, 1971

#### Haliclona (Rhizoniera) rhizophora

(Vacelet, 1969)

#### Haliclona (Rhizoniera) sarai

(Pulitzer-Finali, 1969)

#### Haliclona (Soestella) implexa

(Schmidt, 1868)

#### Haliclona (Soestella) mamillata

(Griessinger, 1971)

#### Haliclona (Soestella) mucosa

(Griessinger, 1971)

#### Niphatidae

#### Pachychalina rustica

Schmidt, 1868

#### Petrosiidae

#### Petrosia (Petrosia) clavata

(Esper, 1794)

#### Petrosia (Petrosia) ficiformis

(Poiret, 1789)

#### Petrosia pulitzeri

Pansini, 1996

#### Phloeodictyidae

#### Calyx nicaeensis

(Risso, 1826)

#### MERLIIDA

#### Hamacanthidae

#### Hamacantha (Vomerula) falcula

(Bowerbank, 1874)

#### Merliidae

#### Merlia normani

Kirkpatrick, 1908

#### POECILOSCLERIDA

#### Coelosphaeridae

#### Forcepia (Leptolabis) luciensis

(Topsent, 1888)

#### Lissodendoryx (Lissodendoryx) isodictyalis

(Carter, 1882)

#### Crambeidae

#### Crambe crambe

(Schmidt, 1862)

#### Crellidae

#### Crella (Pytheas) fusifera

Sarà, 1969

#### Crella (Pytheas) sigmata

Topsent, 1925

#### Esperiopsidae

#### Ulosa stuposa

(Esper, 1794)

#### Ulosa tenellula

Pulitzer-Finali, 1983

#### Hymedesmiidae

#### Hemimycale columella

(Bowerbank, 1874)

#### Hymedesmia (Hymedesmia) pansa

Bowerbank, 1882

#### Hymedesmia (Hymedesmia) peachii

Bowerbank, 1882

#### Hymedesmia (Hymedesmia) simillima

Lundbeck, 1910

#### Hymedesmia (Hymedesmia) versicolor

(Topsent, 1893)

#### Phorbas fictitius

(Bowerbank, 1866)

#### Phorbas posidoni

Voultsiadou-Koukoura & van Soest, 1991

#### Phorbas tenacior

(Topsent, 1925)

#### Phorbas topsenti

Vacelet & Perez, 2008

#### Microcionidae

#### Antho (Antho) involvens

(Schmidt, 1864)

#### Clathria (Clathria) toxistricta

Topsent, 1925

#### Clathria (Clathria) toxistyla

(Sarà, 1959)

#### Clathria (Clathria) toxivaria

(Sarà, 1959)

#### Clathria (Microciona) cleistochela

(Topsent, 1925)

#### Clathria (Microciona) gradalis

Topsent, 1925

#### Clathria (Thalysias) jolicoeuri

(Topsent, 1892)

#### Echinoclathria translata

(Pulitzer-Finali, 1978)

#### Mycalidae

#### Mycale (Aegogropila) contarenii

(Lieberkühn, 1859)

#### Mycale (Aegogropila) retifera

Topsent, 1924

#### Mycale (Aegogropila) rotalis

(Bowerbank, 1874)

#### Mycale (Aegogropila) syrinx

(Schmidt, 1862)

#### Mycale (Aegogropila) tunicata

(Schmidt, 1862)

#### Mycale (Carmia) macilenta

(Bowerbank, 1866)

#### Mycale (Mycale) lingua

(Bowerbank, 1866)

#### Mycale (Mycale) massa

(Schmidt, 1862)

#### Mycale (Paresperella) serrulata

Sarà & Siribelli, 1960

#### Myxillidae

#### Myxilla (Myxilla) rosacea

(Lieberkühn, 1859)

#### Tedaniidae

#### Tedania (Tedania) anhelans

(Vio in Olivi, 1792)

#### POLYMASTIIDA

#### Polymastiidae

#### Tentorium levantinum

Ilan, Gugel, Galil & Janussen, 2003

#### Weberella verrucosa

Vacelet, 1960

#### SCOPALINIDA

#### Scopalinidae

#### Scopalina lophyropoda

Schmidt, 1862

#### SPONGILLIDA

#### Spongillidae

#### Ephydatia fluviatilis

(Linnaeus, 1759)

#### Eunapius carteri

(Bowerbank, 1863)

#### SUBERITIDA

#### Halichondriidae

#### Axinyssa aurantiaca

(Schmidt, 1864)

#### Axinyssa michaelis

Kefalas & Castritsi-Catharios, 2007

#### Halichondria (Halichondria) contorta

(Sarà, 1961)

#### Halichondria (Halichondria) panicea

(Pallas, 1766)

#### Hymeniacidon perlevis

(Montagu, 1814)

#### Laminospongia subtilis

Pulitzer-Finali, 1983

#### Spongosorites flavens

Pulitzer-Finali, 1983

#### Spongosorites intricatus

(Topsent, 1892)

#### Topsentia vaceleti

Kefalas & Castritsi-Catharios, 2012

#### Stylocordylidae

#### Stylocordyla pellita

(Topsent, 1904)

#### Suberitidae

#### Aaptos aaptos

(Schmidt, 1864)

#### Aaptos papillata

(Keller, 1880)

#### Protosuberites denhartogi

van Soest & de Kluijver, 2003

#### Protosuberites ectyoninus

(Topsent, 1900)

#### Protosuberites rugosus

(Topsent, 1893)

#### Pseudosuberites sulphureus

(Bowerbank, 1866)

#### Rhizaxinella pyrifera

(Delle Chiaje, 1828)

#### Rhizaxinella shikmonae

Ilan, Gugel, Galil & Janussen, 2003

#### Suberites carnosus

(Johnston, 1842)

#### Suberites domuncula

(Olivi, 1792)

#### Suberites ficus

(Johnston, 1842)

#### Suberites massa

Nardo, 1847

#### Suberites syringella

(Schmidt, 1868)

#### Terpios gelatinosa

(Bowerbank, 1866)

#### TETHYIDA

#### Hemiasterellidae

#### Hemiasterella aristoteliana

Voultsiadou-Koukoura & van Soest, 1991

#### Tethyidae

#### Tethya aurantium

(Pallas, 1766)

#### Tethya citrina

Sarà & Melone, 1965

#### Timeidae

#### Timea chondrilloides

(Topsent, 1904)

#### Timea geministellata

Pulitzer-Finali, 1978

#### Timea stellata

(Bowerbank, 1866)

#### Timea stellifasciata

Sarà & Siribelli, 1960

#### Timea unistellata

(Topsent, 1892)

#### TETRACTINELLIDA

#### Ancorinidae

#### Dercitus (Stoeba) plicatus

(Schmidt, 1868)

#### Jaspis johnstonii

(Schmidt, 1862)

#### Stelletta dorsigera

Schmidt, 1864

#### Stelletta grubii

Schmidt, 1862

#### Stelletta hispida

(Buccich, 1886)

#### Stelletta mediterranea

(Topsent, 1893)

#### Stelletta stellata

Topsent, 1893

#### Stryphnus (Stryphnus) mucronatus

(Schmidt, 1868)

#### Stryphnus (Stryphnus) ponderosus

(Bowerbank, 1866)

#### Azoricidae

#### Leiodermatium (Leiodermatium) lynceus

Schmidt, 1870

#### Calthropellidae

#### Calthropella (Calthropella) pathologica

(Schmidt, 1868)

#### Calthropella (Corticellopsis) stelligera

(Schmidt, 1868)

#### Geodiidae

#### Erylus discophorus

(Schmidt, 1862)

#### Geodia conchilega

Schmidt, 1862

#### Geodia cydonium

(Jameson, 1811)

#### Penares euastrum

(Schmidt, 1868)

#### Penares helleri

(Schmidt, 1864)

#### Pachastrellidae

#### Pachastrella monilifera

Schmidt, 1868

#### Samidae

#### Samus anonymus

Gray, 1867

#### Tetillidae

#### Craniella cranium

(Müller, 1776)

#### Theneidae

#### Thenea muricata

(Bowerbank, 1858)

#### Theonellidae

#### Discodermia polymorpha

Pisera & Vacelet, 2011

#### Thoosidae

#### Alectona millari

Carter, 1879

#### Vulcanellidae

#### Poecillastra compressa

(Bowerbank, 1866)

#### Vulcanella gracilis

(Sollas, 1888)

#### VERONGIIDA

#### Aplysinidae

#### Aplysina aerophoba

(Nardo, 1833)

#### Aplysina cavernicola

(Vacelet, 1959)

#### Ianthellidae

#### Hexadella pruvoti

Topsent, 1896

#### Hexadella racovitzai

Topsent, 1896

#### HEXACTINELLIDA

#### LYSSACINOSIDA

#### Rossellidae

#### Sympagella nux

Schmidt, 1870

#### HOMOSCLEROMORPHA

#### HOMOSCLEROPHORIDA

#### Oscarellidae

#### Oscarella balibaloi

Pérez, Ivanisevic, Dubois, Pedel, Thomas, Tokina & Ereskovsky, 2011

#### Oscarella lobularis

(Schmidt, 1862)

#### Oscarella microlobata

Muricy, Boury-Esnault, Bézac & Vacelet, 1996

#### Oscarella tuberculata

(Schmidt, 1868)

#### Pseudocorticium jarrei

Boury-Esnault, Muricy, Gallissian & Vacelet, 1995

#### Plakinidae

#### Corticium candelabrum

Schmidt, 1862

#### Plakina bowerbanki

(Sarà, 1960)

#### Plakina dilopha

Schulze, 1880

#### Plakina monolopha

Schulze, 1880

#### Plakina trilopha

Schulze, 1880

#### Plakina weinbergi

Muricy, Boury-Esnault, Bézac & Vacelet, 1998

#### Plakinastrella copiosa

Schulze, 1880

#### Plakortis simplex

Schulze, 1880

## Discussion

A total of 215 species, classified to 111 genera, 65 families, 24 orders, and 4 classes makes the updated checklist of Porifera of Greece. Demosponges and Homoscleromorpha make up the bulk of Porifera of Greece, while only 8 species of Calcarea are listed for the first time from the area. As it can be seen from the list, the orders Poecilosclerida, Dictyoceratida, Tetractinellida, Haplosclerida, and Suberitida have the highest number of species comprising 62% of the known Greek sponge species richness.

The majority of species included in the present checklist were already known as elements of the Greek fauna (Voultsiadou 2005a), while 34 new additions were made in the course of this study (Table 1). These additions include 8 species of Calcarea, 17 species of Demospongiae, 1 species of Hexactinellida, and 6 species of Homoscleromorpha. Two more, freshwater species, *Ephydatia fluviatilis* and *Eunapius carteri*, were also added as elements of the Greek fauna.

It is worth mentioning that 8 of the species included in the list (*Axinyssa michaelis, Coscinoderma sporadense, Hemiasterella aristoteliana, Ircinia paucifilamentosa, Leucandra nausicaae*, *Petrosia pulitzeri, Phorbas posidoni*, and *Topsentia vaceleti*) have been first described from Greek waters. All except one (*P. pulitzeri*) are considered endemic to this area, since they have not been yet reported elsewhere. A number of species, endemic to the eastern Mediterranean, have been also recorded in Greek waters, such as the deep-sea sponges *Sycon faulkneri*, *Rhizaxinella shikmonae*, and *Tentorium levantinum*, which were described from the Levantine, but were also recorded from the Sea of Crete and Sporades Basin by Ilan et al. (2003). Interestingly, most of the aforementioned species were found in dimly lit habitats which can be sponge-dominated (Gerovasileiou and Voultsiadou 2012), such as marine caves, coralligenous beds, and the bathyal zone, highlighting the need for further research to reveal the unknown diversity in these environments.

The list of sponges of Greece compiled for the needs of the present study is the most comprehensive list of Porifera in the Eastern Mediterranean. There is another list presenting 131 sponge species from the Turkish coasts (Topaloğlu and Evcen 2014) and various scattered sources of information on the sponges of the remaining Levantine coasts (see Voultsiadou 2005b). However, a major gap of our knowledge on the sponge species richness from the Greek seas is obvious since practically the entire bibliography on Greek sponges concerns the Aegean Sea, while very few and scattered information is available from the Ionian coasts of Greece (e.g. Schuffner 1877, Laborel 1960). Finally, it should be mentioned that several species reported from the Turkish coasts of the Aegean have not been yet found in Greek waters. Given that both Turkish and Greek coasts are part of the broader ecoregion of the Aegean Archipelago (Spalding et al. 2007), it is reasonable to assume that it is a matter of time before these species are recorded as elements of the Greek fauna as well.

## Supplementary Material

Supplementary material 1 Checklist of Porifera of Greece Data type: Taxonomic checklist
Brief description: Taxonomic checklist of Porifera known to occur in Greek waters.
File: oo_98685.xls Eleni Voultsiadou, Vasilis Gerovasileiou, Nicolas Bailly

## Figures and Tables

**Table 1.** Marine sponge species added by the present study (not included in the list given by Voultsiadou 2005a). For each species the publication mentioning its occurrence in the Greek seas is given.

**Species**	**References**
Class **Calcarea**	
*Clathrina blanca* (Miklucho-Maclay, 1868)	Longo and Pronzato (2011)
*Clathrina clathrus* (Schmidt, 1864)	Gerovasileiou et al. (2015)
*Leucandra nausicaae* (Schuffner, 1877)	Burton (1963)
*Leucetta solida* (Schmidt, 1862)	Pansini et al. (2000)
*Sycon elegans* (Bowerbank, 1845)	Gerovasileiou et al. (2015)
*Sycon faulkneri* Ilan, Gugel, Galil & Janussen, 2003	Ilan et al. (2003)
*Sycon raphanus* Schmidt, 1862	Tortonese (1947)
*Petrobiona massiliana* Vacelet & Lévi, 1958	Hermans et al. (2009)
Class **Demospongiae**	
*Axinyssa michaelis* Kefalas & Castritsi-Catharios, 2007	Kefalas and Castritsi-Catharios (2007)
Clathria (Clathria) toxistricta Topsent, 1925	Voultsiadou et al. (2010)
*Cliona parenzani* Corriero & Scalera-Liaci, 1997	Vacelet et al. (2008)
Forcepia (Leptolabis) luciensis (Topsent, 1888)	Voultsiadou et al. (2010)
Haliclona (Gellius) microsigma (Babic, 1922)	Gerovasileiou and Voultsiadou (2012)
Haliclona (Halichoclona) perlucida (Griessinger, 1971)	Gerovasileiou and Voultsiadou (2012)
*Hexadella pruvoti* Topsent, 1896	Gerovasileiou and Voultsiadou (2012)
*Hexadella racovitzai* Topsent, 1896	Gerovasileiou and Voultsiadou (2012)
Hymedesmia (Hymedesmia) pansa Bowerbank, 1882	Voultsiadou et al. (2010)
*Hyrtios collectrix* (Schulze, 1880)	Voultsiadou et al. (2010)
*Ircinia retidermata* Pulitzer-Finali & Pronzato, 1981	Bianchi et al. (2014)
*Petrosia pulitzeri* Pansini, 1996	Pansini (1996)
*Protosuberites rugosus* (Topsent, 1893)	Gerovasileiou and Voultsiadou (2012)
*Rhizaxinella sikmonae* Ilan, Gugel, Galil & Janussen, 2003	Ilan et al. (2003)
*Tentorium levantinum* Ilan, Gugel, Galil & Janussen, 2003	Ilan et al. (2003)
*Thymosiopsis cuticulatus* Vacelet & Perez, 1998	Gerovasileiou and Voultsiadou (2012)
*Topsentia vaceleti* Kefalas & Castritsi-Catharios, 2012	Kefalas and Castritsi-Catharios (2012)
Class **Hexactinellida**	
*Sympagella nux* Schmidt, 1870	See Boury-Esnault et al. (2014)
Class **Homoscleromorpha**	
*Oscarella balibaloi* Pérez et al., 2011	Gerovasileiou and Voultsiadou (2012)
*Oscarella microlobata* Muricy et al., 1996	Gerovasileiou and Voultsiadou (2012)
*Oscarella tuberculata* (Schmidt, 1868)	Gerovasileiou and Voultsiadou (2012)
*Plakina bowerbanki* (Sarà, 1960)	Gerovasileiou and Voultsiadou (2012)
*Plakina weinbergi* Muricy et al., 1998	Ereskovsky et al. (2009)
*Pseudocorticium jarrei* Boury-Esnault et al., 1995	Gerovasileiou and Voultsiadou (2012)
